# Disentangling Temporal Trends of Clade Ib Monkeypox Virus Transmission in Burundi

**DOI:** 10.1093/infdis/jiaf475

**Published:** 2025-09-10

**Authors:** Shihui Jin, Toshiaki R Asakura, Hiroaki Murayama, Sung-mok Jung, David Niyukuri, Joseph Nyandwi, Liliane Nkengurutse, Olivier Kamatari, Jue Tao Lim, Akira Endo, Borame L Dickens

**Affiliations:** Saw Swee Hock School of Public Health, National University of Singapore and National University Health System, Singapore, Singapore; Department of Infectious Disease Epidemiology and Dynamics, London School of Hygiene & Tropical Medicine, London, United Kingdom; Centre for Mathematical Modelling of Infectious Diseases, London School of Hygiene & Tropical Medicine, London, United Kingdom; School of Tropical Medicine and Global Health, Nagasaki University, Nagasaki, Japan; Department of Infectious Disease Epidemiology and Dynamics, Institute of Tropical Medicine, Nagasaki University, Nagasaki, Japan; School of Medicine, International University of Health and Welfare, Narita, Japan; Saw Swee Hock School of Public Health, National University of Singapore and National University Health System, Singapore, Singapore; Carolina Population Centre, University of North Carolina at Chapel Hill, Chapel Hill, North Carolina, USA; Doctoral School, University of Burundi, Bujumbura, Burundi; Department of Mathematics, University of Burundi, Bujumbura, Burundi; Centre of Excellence in Epidemiological Modelling and Analysis (SACEMA) and Centre for Epidemic Response and Innovation (CERI), Stellenbosch University, Cape Town, South Africa; National Institute of Public Health, Bujumbura, Burundi; Faculty of Medicine, University of Burundi, Bujumbura, Burundi; Doctoral School, University of Burundi, Bujumbura, Burundi; Public Health Emergency Operation Centre, Bujumbura, Burundi; Doctoral School, University of Burundi, Bujumbura, Burundi; Public Health Emergency Operation Centre, Bujumbura, Burundi; Lee Kong Chian School of Medicine, Nanyang Technological University, Singapore, Singapore; Saw Swee Hock School of Public Health, National University of Singapore and National University Health System, Singapore, Singapore; School of Tropical Medicine and Global Health, Nagasaki University, Nagasaki, Japan; Department of Infectious Disease Epidemiology and Dynamics, Institute of Tropical Medicine, Nagasaki University, Nagasaki, Japan; Saw Swee Hock School of Public Health, National University of Singapore and National University Health System, Singapore, Singapore

**Keywords:** Burundi, clade Ib, monkeypox virus, mpox, transmission

## Abstract

Utilizing mpox case data from Burundi between August 2024 and April 2025, we calibrated a mathematical model to quantify the temporal trends of clade Ib monkeypox virus transmission. The model outputs indicated a declining overall transmission trend. Children aged 0–4 and 5–9 years were estimated to be at higher risk of infection compared to older age groups, while sexual contact was inferred to contribute up to 50% of the overall transmission.

## Teaser

We modeled the transmission dynamics of clade Ib monkeypox virus in Burundi using case data from August 2024 to April 2025. Our estimates suggest an overall decline but persistently elevated infection risks among children under 10 years and high-sexual-activity individuals.

A novel subclade of clade I monkeypox virus (MPXV), named clade Ib, was first identified in South Kivu, Democratic Republic of Congo (DRC) in September 2023 [1]. Since then, the virus has spread to other provinces in the DRC and reached the neighboring country of Burundi on 25 July 2024. This led to a large-scale outbreak in the local community [1], where 3839 confirmed cases were reported in Burundi as of 27 April 2025 [2]. Transmission has occurred through both sexual and nonsexual close contact [3], with a substantial proportion of confirmed cases reported among children aged under 10 years and young adults aged 20–29 years, and approximately half being females [4]. Here, we estimated the time-varying transmission trend of clade Ib MPXV in Burundi from August 2024 to April 2025 and investigated potential factors associated with the temporal case trends, including sexual transmission, adult-to-child transmission, and infection risk of children, to provide epidemiological insights into the current outbreak.

## METHODS

Weekly case counts stratified by age and sex were obtained from daily situation reports published by Burundi Public Health Emergency Operation Centre (Ministry of Health) [4], covering the period from 18 August (Epidemiological week [Eweek] 33) 2024 to 20 April (Eweek 16) 2025. We assumed that all the cases were clade Ib infections, given the lack of evidence suggesting circulation of other MPXV clades in the country [2]. These data were used to calibrate a next generation matrix (NGM) model adapted from a previous study [5]. The model characterized transmission patterns of clade Ib MPXV by stratifying the infections by age, sex, sexual activity level, and route of exposure (sexual vs. community), and by parameterizing the expected number of onward transmissions from each case to each subpopulation in a matrix format [1]. We assumed that 10% of individuals aged 15–49 years (the assumed sexually active age group) were highly sexually active, engaging in higher rates of sexual contact than those represented in the empirical community contact matrix, with this proportion varied in a sensitivity analysis. Children aged 0–4 and 5–9 years were presumed to have distinct infection risks per infectious contact compared to the general community [1, 6]. Meanwhile, people aged 45 years and above, who were born before smallpox was declared eradicated in 1980 [7], were assumed to have partial protection from historical smallpox vaccination [8].

Temporal variations were further introduced to the NGM ($M_{t}$) to capture the evolving transmission dynamics. We assumed that community mixing patterns remained constant over time, while allowing for changes in: (1) the scaling of the next generation matrix, which reflects the overall temporal trends in transmission of clade Ib MPXV, (2) the relative infection risk per infectious contact for children aged 0–4 and 5–9 years to the remaining general population, and (3) the sexual contact rates for high-sexual-activity males and females, defined as the amount of sexual contact relative to one unit of daily community contact leading to disease transmission. These variations were smoothed over time using spline interpolation. The expected number of incidence in week *t* (stratified by age, sex, and sexual activity level), $I_{t}$, was then modeled by


$$EI_{t}=M_{t}\underset{\tau =1,2,\cdots ,t-1}{\sum }\;g_{\tau }I_{t-\tau },$$

where $\left\{g_{t}\right\}$ corresponds to the discretized serial interval distribution. It was estimated from symptom onset dates of household infector–infectee pairs during historical clade Ia outbreaks in Sudan and Central African Republic based on published data [9]. The largest eigenvalue of $M_{t}$ generated the instantaneous reproduction number, $R_{t}$, which quantifies disease transmission potential at time *t* [10]. The observed case count for individuals of age *a* and sex *s* in week *t* was assumed to follow a Poisson distribution, with the mean equal to the total weekly incidence of age *a* and sex *s*, aggregated across sexual activity levels where applicable.

Within a Bayesian framework, we employed a Non-U-Turn sampler to estimate the aforementioned temporal parameters, along with time-invariant smallpox vaccine effectiveness against clade Ib infection and proportions of high-sexual-activity males and females within individual age groups. Flat prior distributions were assigned to all parameters except for the smallpox vaccine effectiveness and the scaling of the NGM. Further details of model parameterization and fitting are provided in the Supplementary Information (Supplementary Table 1).

## RESULTS

**Figure 1.**
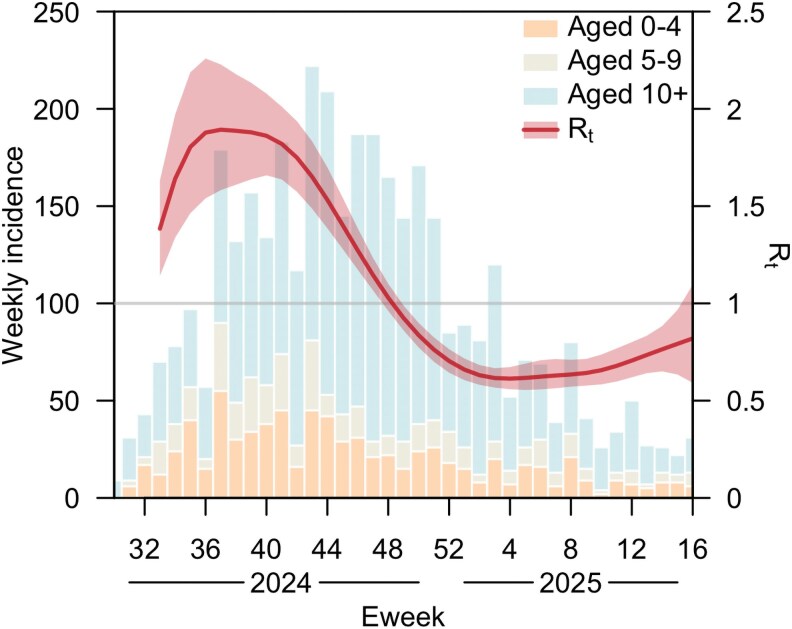
Clade Ib incidence and $R_{t}$ estimates for Burundi. The bars represent reported weekly case counts for children aged 0–4 (orange, bottom), 5–9 (yellow, middle), and all other age groups (blue, top) [4]. The red curve shows the posterior mean of $R_{t}$, with shaded area indicating the corresponding 95% credible intervals. The gray solid line represents the threshold value of one for $R_{t}$. The time frame for case data spans from 28 July (Eweek 30) 2024 to 20 April (Eweek 16) 2025, while that for $R_{t}$ estimates spans from 18 August 2024 (Eweek 33) to 20 April 2025 (Eweek 16).

The estimated $R_{t}$ exhibited a generally downward trend, declining substantially from 1.89 (95% credible interval [CrI]: 1.58–2.23) in Eweek 37 (15 September 2024), crossing the epidemic threshold of one in Eweek 48 (1 December 2024), and stabilizing at approximately 0.65 in the first quarter of 2025 (Figure 1). This pattern remained consistent across various model parameter settings, including degrees of smoothing, prior specifications for the scaling factor, serial interval distribution, high-sexual-activity population size, constraints on temporal parameters, parameterizations of sexual mixing patterns, and assumed distribution of weekly case counts (Supplementary Figures 3–8 and 10). The posterior predictive distribution of case counts generated by the model fits the observed data well (Supplementary Figures 2 and 9).

**Figure 2.**
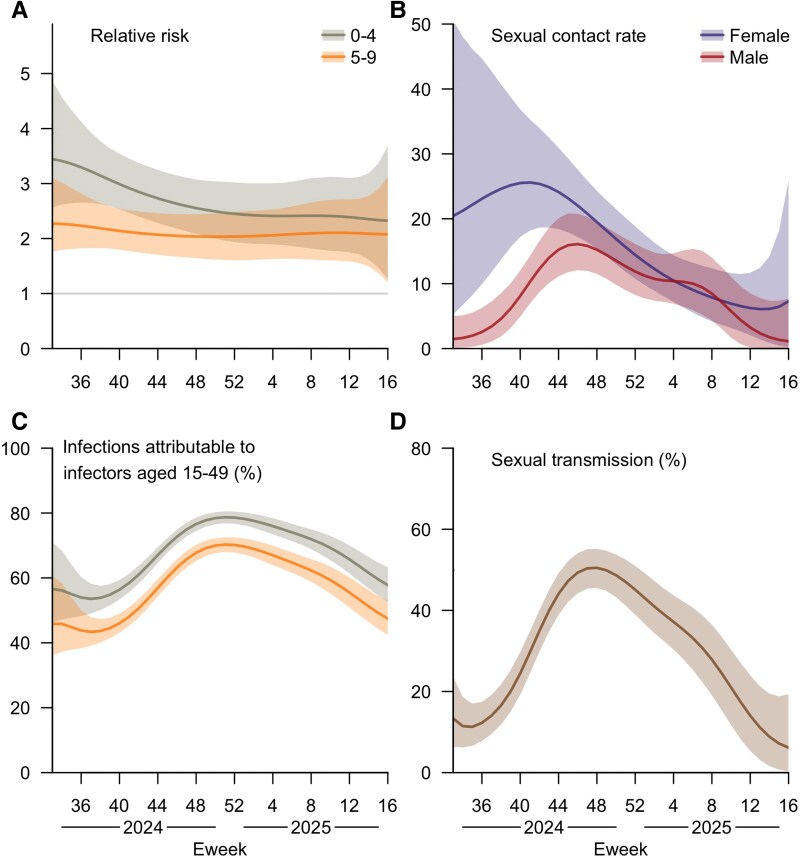
Inferred temporal outbreak profile for children and high-sexual-activity individuals. These include (*A*) the relative infection risk per infectious contact for children aged 0–4 and 5–9 y compared to the remaining general population, (*B*) the sexual contact rates of the high-sexual-activity groups, (*C*) the proportion of infections among children aged 0–4 and 5–9 y attributable to infectors aged 15–49 y, and (*D*) the proportion of transmission through sexual contact. All the estimates are presented as posterior means (lines) with 95% credible intervals (shaded areas). The time frame spans from 18 August (Eweek 33) 2024 to 20 April (Eweek 16) 2025.

Both the 0–4 and 5–9 year age groups were estimated to be at significantly higher risks of infection compared to the general population, with those aged 0–4 years being consistently more likely to acquire mpox throughout the inference period. For instance, the inferred relative risks were 3.45 (95% CrI: 2.56–4.86) for children aged 0–4 years and 2.27 (95% CrI: 1.76–3.10) for those aged 5–9 years in Eweek 33 (18 August) of 2024, gradually declining to 2.32 (95% CrI: 1.26–3.68) and 2.08 (95% CrI: 1.21–3.12), respectively, for these two age groups in Eweek 16 (20 April) of 2025 (Figure 2*A*). Most infections in this youngest age group were inferred to be acquired from individuals aged 15–49 years (ie, the assumed sexually active age group), who accounted for up to 78.7% (95% CrI: 76.8%–80.5%) of the infections aged 0–4 years at the peak in Eweek 51 (22 December 2024; Figure 2*A* and *C*).

Alongside the inferred rising contact rates among high-sexual-activity individuals during the early stages of the outbreak, the proportion of transmission through sexual contact, defined as the expected fraction of new infections acquired through sexual transmission among all incidence, was estimated to increase from 11% (95% CrI: 7%–17%) in Eweek 35 (8 September) of 2024 to 51% (95% CrI: 46%–55%) at a peak around Eweek 48 (1 December 2024), before declining to 6% (95% CrI: 0.3%–19%) in Eweek 16 (20 April) of 2025. In addition, the estimated rate of sexual contact from high-sexual-activity females to males was greater than vice versa, although the disparity diminished by early 2025 (Figure 2*B* and *D*).

## DISCUSSION

Our analysis provides an update of the evolving epidemiological landscape of clade Ib MPXV transmission in Burundi from August 2024 to April 2025, suggesting a generally nonincreasing trend in disease transmission and consistently greater transmission or infection risks among key subpopulations, namely young children and high-sexual-activity individuals.

The inferred decrease in mpox transmission within the general population might be attributed to a reduction in the frequency of close contacts as public awareness of the disease increased and sanitary practices improved. Another plausible explanation matching our estimated trend is that the early phase of the outbreak was driven by individuals with high connectivity with others, and that the transmission potential subsequently declined with the depletion of susceptibles in this subpopulation.

Our findings of higher per-contact infection risks among children aged under 10 years might be partially explained by their frequent and prolonged close contact with parents or caregivers, who are usually aged 15–49 years. The potential for MPXV transmission via fomites could also contribute to this elevated risk, particularly in resource-limited settings where co-sleeping, shared-living spaces, and communal use of household items are common [1]. These conditions are especially relevant for very young children under five years old, who require closer contact with adults and may potentially come into contact with lesions [11].

There are a few limitations worth highlighting. Firstly, we approximated community mixing patterns using empirical home contact data, substantiated by the reported predominance of household-based transmission aside from sexual routes [1]. While this may overlook nonhousehold and fomite-mediated transmission, we adjusted for contact variation over time and across age groups by incorporating a time-varying scaling parameter and age-specific infection risks. Additionally, in the absence of empirical data from Burundi, we utilized contact survey data from Zimbabwe [12], adjusted for age distribution. This proxy of contact behaviors in Burundian households is supported by similarities in average household size (5–6 individuals) and age composition of household members [13], as well as geographical proximity and predominant Christian religious affiliation in both countries. A principal component analysis of characteristics potentially relevant to contact patterns further indicated high similarities between the two countries [5].

Secondly, we utilized the serial interval distribution from historical clade Ia outbreaks in nearby countries [9] as a proxy for clade Ib transmission in Burundi due to the absence of relevant estimates for clade Ib in the literature as of April 2025. Among the two available distributions derived by Marziano et al. from household- and hospital-based outbreaks [9], we opted for the household-based distribution in the main analysis (with the hospital-based distribution assessed in the sensitivity analysis) because of the predominance of infections in household settings in Burundi [1]. It should be noted that this distribution might not well represent the 2024–2025 clade Ib outbreak, due to the limited sample size of the source data, contextual differences such as the countries affected, as well as the emergence of heterosexual transmission as an important transmission route [1]. Nevertheless, the sensitivity analysis employing an alternative serial interval distribution with smaller variation suggested similar temporal trend estimates (Supplementary Figure 5).

Thirdly, under-reporting was not explicitly modeled due to a lack of reliable indicators. Although $R_{t}$ estimates would remain robust assuming a time-invariant case-ascertainment rate [10], limited early-stage surveillance (especially among young adults) suggests potential fluctuations in reporting [14]. With the progression of the outbreak, growing public awareness of mpox encouraged more symptomatic infections to seek medical assistance, while the identification of sexual transmission as a key route prompted local authorities to expand targeted testing among key populations (eg, sex workers) [1]. These changes may have increased the case-ascertainment rate over time, potentially affecting temporal trends of the inferred transmission dynamics. At the same time, they also highlight the value of analyses like this study in quantifying evolving subpopulation-specific infection risks and informing timely, targeted surveillance and containment efforts.

Fourthly, the onset-to-reporting delays were not incorporated into our model owing to insufficient data to quantify their extent, but substantial or variable delays may affect reliability of model estimates. Furthermore, while spline smoothing mitigated the impact of short-term fluctuations in reported case counts, the limited number of data points at the beginning and end of the study period led to greater uncertainty in the parameter estimates for those time intervals (Figures 1 and 2).

Lastly, importation was not considered in the model due to the absence of relevant data and prior evidence of local transmission dominance [1]. Nevertheless, given the substantial cross-border movements from the DRC [15], including refugee influxes and trade, this omission may have resulted in an overestimation of the transmission potential.

## Supplementary Material

jiaf475_Supplementary_Data

## Supplementary Data

Supplementary materials are available at *The Journal of Infectious Diseases* online (http://jid.oxfordjournals.org/). Supplementary materials consist of data provided by the author that are published to benefit the reader. The posted materials are not copyedited. The contents of all supplementary data are the sole responsibility of the authors. Questions or messages regarding errors should be addressed to the author.
