# Supplementary material for: Disentangling Temporal Trends of Clade Ib Monkeypox Virus Transmission in Burundi
Source: J Infect Dis. 2025 Sep 10;233(1):e140–4. doi: 10.1093/infdis/jiaf475 (PMC12811874; doi:10.1093/infdis/jiaf475)
Supplement: jiaf475_Supplementary_Data [file jiaf475_supplementary_data.pdf]

Supplementary Information

Methods 2
Model fit of the main analysis 7
Sensitivity analysis: Impact of spline function degree of freedom on transmission trend estimates 8
Sensitivity analysis: Impact of prior distribution for the scaling of the NGM on transmission trend estimates 10
Sensitivity analysis: Impact of serial interval distribution on transmission trend estimates 11
Sensitivity analysis: Impact of high-sexual-activity population size on transmission trend estimates 12
Sensitivity analysis: Impact of temporal constraints on transmission trend estimates 13
Sensitivity analysis: Impact of sexual mixing pattern on transmission trend estimates 15
Sensitivity analysis: Impact of reported case distribution assumption on transmission trend estimates 16
References 18

Methods

Summary of the next generation matrix model

We adapted an existing model based on the next generation matrix (NGM) developed elsewhere [1] to incorporate temporal dynamics. Full details of the model settings can be found in the original study.

The population was divided into eight age groups: 0–4, 5–9, 10–14, 15–19, 20–29, 30–39, 40–49, and 50+, following those specified in the situation reports. Each age group was further stratified by sex and sexual activity level (low and high, with high-sexual-activity individuals limited to 15–49 years). The NGM at time t, $\boldsymbol{M}_t$, equalled the product of a scaling factor (c_t), group-specific susceptibility, and a contact matrix incorporating both community and sexual contact, described as below in the block-matrix format:

$$c_t \cdot \begin{pmatrix} \boldsymbol{D}_t & \boldsymbol{O} & \boldsymbol{O} & \boldsymbol{O} \\ \boldsymbol{O} & \boldsymbol{D}_t & \boldsymbol{O} & \boldsymbol{O} \\ \boldsymbol{O} & \boldsymbol{O} & \boldsymbol{D}_t & \boldsymbol{O} \\ \boldsymbol{O} & \boldsymbol{O} & \boldsymbol{O} & \boldsymbol{D}_t \end{pmatrix} \begin{pmatrix} \boldsymbol{O} & \boldsymbol{O} & \boldsymbol{S}_{t,MF} & \boldsymbol{\Sigma}_{t,MF}\boldsymbol{Q}_F \\ \boldsymbol{C}_{MM} & \boldsymbol{C}_{MM} & \boldsymbol{C}_{MF} & \boldsymbol{C}_{MF} \\ \boldsymbol{S}_{t,FM} & \boldsymbol{\Sigma}_{t,FM}\boldsymbol{Q}_M & \boldsymbol{O} & \boldsymbol{O} \\ \boldsymbol{C}_{FM} & \boldsymbol{C}_{FM} & \boldsymbol{C}_{FF} & \boldsymbol{C}_{FF} \end{pmatrix}, \quad (1)$$

where $\boldsymbol{O}$ is a zero matrix.

Group-specific infection risk was represented by the block diagonal matrix in (1). Under the assumption of uniform smallpox vaccine coverage across the two sexual activity levels, this matrix comprised four identical diagonal blocks, each donated as $\boldsymbol{D}_t = \mathrm{diag}(d_t)$, capturing age-specific risks. This included differentiated per-contact infection risks among children aged 0–4 (σ_t^{0-4}) and 5–9 years (σ_t^{5-9}), which accounted for factors related to susceptibility and levels of close contact not represented by the community contact matrix. We also accounted for reduced susceptibility (by e_s) due to immunity from historical smallpox vaccination, i.e.,

$$d_{t,a} = \begin{cases} \sigma_t^{0-4}, 0 \le a \le 4 \\ \sigma_t^{5-9}, 5 \le a \le 9 \\ 1, 10 \le a \le 39 \\ 1 - e_s/2, 40 \le a \le 49 \\ 1 - e_s, a \ge 50 \end{cases}. \quad (2)$$

Following Taube et al. [2] and the cessation of mass smallpox vaccination after the smallpox eradication in 1980 [3], we assumed half of the 40–49-year-old individuals and all of those aged 50 years and above might have some protection against Clade Ib MPXV. However, immunity from previous mpox infection or mpox-specific vaccines was not accounted for, since Burundi was mpox-free before the 2024–2025 mpox outbreak and had no documented mass mpox vaccination campaigns [4].

Let the subscripts M and F represent males and females, respectively. The community contact matrix, $\boldsymbol{C}_X$, in the block contact matrix (1) was assumed to be time-invariant and derived from the home contact data in the Zimbabwe contact survey [5], which well

captured the mixing patterns relevant to clade I MPXV transmission in the general community [1]. We further adjusted this empirical matrix using demographic data from the 2012 Zimbabwe (Manicaland) census and the 2024 Burundi census. Specifically, let $\boldsymbol{C}' = (c'_{ab})$ represent the empirical Zimbabwe contact matrix, with element c'_{ab} denoting the average number of contacts in age group b from an individual in age group a. Then the contact matrix for Burundi (unstratified by sex), $\boldsymbol{C} = (c_{ab})$, was calculated as

$$c_{ab} = c'_{ab} \times \frac{n_b^B}{n_b^Z} \times \frac{N^Z}{N^B}, \quad (3)$$

where n_a^Z and n_a^B were the sizes of age group a in Zimbabwe and Burundi, respectively, and $N^Z = \sum_a n_a^Z$ and $N^B = \sum_a n_a^B$ were the total population sizes of the two countries (Figure S1). The sex-specific contact matrix, $\boldsymbol{C}_X$, was subsequently derived based on the male and female distribution for each age group.

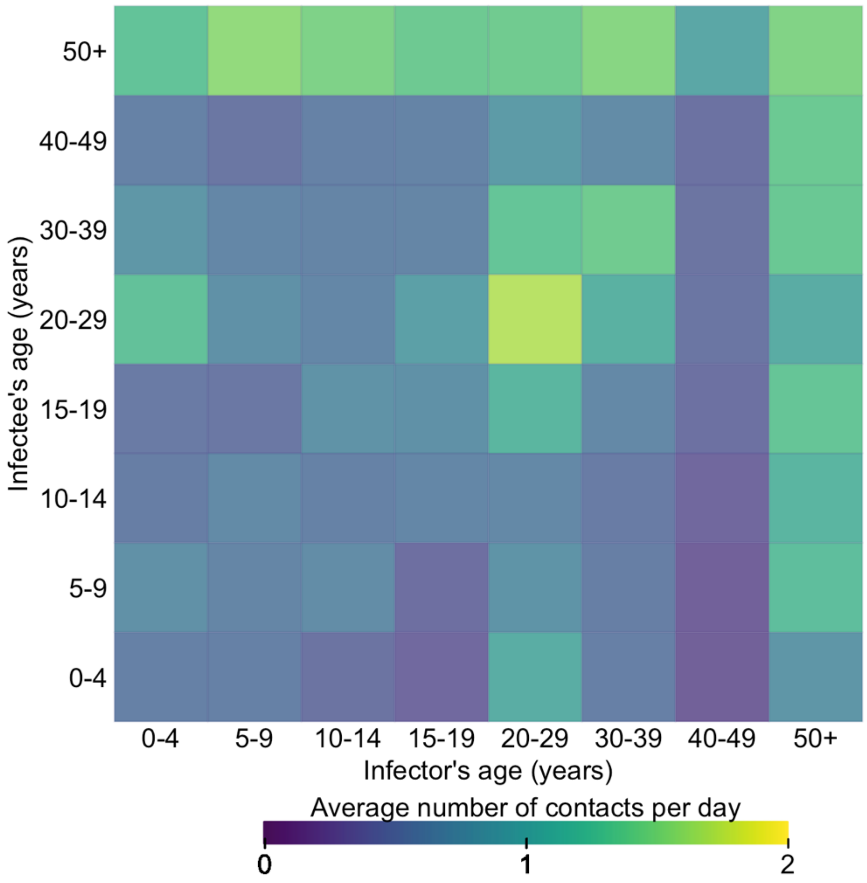

Figure S1. Home contact matrix of Burundi. The heatmap shows the average number of daily household contacts between an infector (column) and an infectee (row) in Burundi, with a lighter (more yellowish) colour representing a higher contact rate.

The heterosexual contacts uncaptured by the home contact matrix were modelled by the time-varying sexual contact matrices $\boldsymbol{S}_{t,X}$ and $\boldsymbol{\Sigma}_{t,X}$. These contacts were attributed to a

subset of highly sexually active individuals aged 15–49 years, who were assumed to engage in sexual activities more frequently than the general population and hence were more likely to acquire and/or transmit the disease through sexual routes. Let the superscripts M and F represent factors associated with sexual transmission for males and females, respectively, while m and f denoted factors related to community transmission. Proportionate mixing patterns were assumed, with the mixing matrices defined as $\left(\boldsymbol{S}_{t,MF}\right)_{ab} = \frac{w_t^F n_a^m q_a^M}{\sum_i n_i^m q_i^M}$ for sexually-acquired female-to-male transmission and $\left(\boldsymbol{S}_{t,FM}\right)_{ab} = \frac{w_t^M n_a^f q_a^F}{\sum_i n_i^f q_i^F}$ for sexually-acquired female-to-male transmission. Here, w_t^F and w_t^M characterized the sexual contact rates of sexually-acquired female and male cases, respectively, defined as the amount of sexual contact contributing to transmission equivalent to one unit of daily community contact. Parameters p_a and q_a denoted the proportions of highly sexually active males and females population, respectively, in age group a which consists of n_a^m males and n_a^f females. In block matrix format, $\boldsymbol{Q}_M = \mathrm{diag}(q_a^M)$ and $\boldsymbol{Q}_F = \mathrm{diag}(q_a^F)$. Collectively, these highly sexually active individuals constituted approximately 10% of the total population aged 15–49 for each sex, with the proportions varied in a subsequent sensitivity analysis.

In addition, $\boldsymbol{S}_{t,X}$ was assumed proportional to $\boldsymbol{\Sigma}_{t,X}$, such that $\boldsymbol{\Sigma}_{t,MF} = \alpha_F \boldsymbol{S}_{t,MF}$ and $\boldsymbol{\Sigma}_{t,FM} = \alpha_M \boldsymbol{S}_{t,FM}$, to account for different sexual transmission rates from sexually-acquired versus community-acquired cases among highly sexually active individuals. This aimed to reflect their differential behaviours where individuals infected through sexual contact were more likely to have high levels of sexual activity and hence tend to transmit the disease via the same routes. The scaling factors α_F and α_M were informed by Inungu et al. [6], and reciprocity was enforced to ensure consistency between female and male sexual contacts:

$$\alpha_F w_F \sum_a n_a^f q_a^F = \alpha_M w_M \sum_a n_a^m q_a^M . \tag{4}$$

It should be noted that the model did not account for sexual transmission between individuals of the same sex, as reported cases showed a balanced gender distribution and there was limited evidence of same-sex sexual transmission as of April 2025 [7–9]. A complete list of model parameters is provided in Table S1. Additional details regarding the model configuration can be found in the original study [1].

Table S1. Parameters and data sources for the NGM model.

Parameter	Definition	Source
c_t	Scaling of the next generation matrix, which reflects the overall temporal trends in transmission of clade Ib MPXV, i.e., a time-dependent proportionality factor	Estimated, using the prior $N^+(0.15, 0.2^2)$ informed by literature [10]
Susceptibility		

σ_t^{0-4}	Time-dependent relative infection risk per infectious contact for children aged 0–4 years compared to the general population	Estimated, using a hierarchical prior $N(\mu_{0-4}, \delta_{0-4}^2)$, where $\mu_{0-4} \sim N(0,10^2)$ and $\delta_{0-4} \sim N^+(0,10^2)$
σ_t^{5-9}	Time-dependent relative infection risk per infectious contact for children aged 5–9 years compared to the general population	Estimated, using a hierarchical prior $N(\mu_{5-9}, \delta_{5-9}^2)$, where $\mu_{5-9} \sim N(0,10^2)$ and $\delta_{5-9} \sim N^+(0,10^2)$
e_s	Effectiveness of smallpox vaccines against mpox infection	Estimated, using the prior $N^+(0.75,0.1^2)$ informed by literature [11]
Contact		
n_a^m	Population size of males in age group a	2024 Burundi census
n_a^f	Population size of females in age group a	2024 Burundi census
p_a^m	Proportion of males within age group a	2024 Burundi census
q_a^M	Proportion of high-sexual-activity males within age group a	Estimated, using a uniform prior lower bounded at 0
q_a^F	Proportion of high-sexual-activity females within age group a	Estimated, using a uniform prior lower bounded at 0
$\boldsymbol{C}_X$	Community contact matrix, approximated through empirical home contact matrix; X can be any of MM, MF, FM, FF, representing interactions within or between males and females	Zimbabwe contact survey [5]
w_t^F, w_t^M	Time-dependent, age-independent sexual contact rates for high-sexual-activity females and males, i.e., the amount contributing to transmission relative to one unit of daily community (home) contact	Estimated, using a uniform prior lower bounded at 0
α_F, α_M	Ratio of number of sexual partners between community- and sexually-acquired infections	Inungu et al. [6]
$\boldsymbol{S}_{t,X}$	Time-dependent sexual contact matrix for interactions between sexually-acquired, high-sexual-activity infections or exposures	Estimated*
$\boldsymbol{\Sigma}_{t,X}$	Time-dependent sexual contact matrix for interactions between sexually-acquired and community-acquired high-sexual-activity infections or exposures	Estimated*

* Constructed from other estimated parameters

Temporal trend smoothing via spline interpolation

To account for gradual variation and minimize overfitting, we modelled the time series of the temporal parameters using B-spline, a piecewise polynomial spline function. Spline functions with six degrees of freedom were selected for the main analysis, which captured the temporal trends in the case data while avoiding overfitting. A sensitivity analysis was further conducted to evaluate the impact of alternative degrees of freedom on temporal trend estimates.

Transmission-related parameter derivation

In addition to the time-varying effective reproduction number, R_t, we calculated the proportion of infections among children aged 0–4 and 5–9 years attributable to infectors aged 5–49 years as $\sum_b m_{t,ab} 1_s(a) \,/\, \sum_b m_{t,ab}$, where $m_{t,ab}$ is the element of $\boldsymbol{M}_t$ with the age group of 0–4 or 5–9 years as the recipient (a, for either males or females), and $1_s(b)$ is an indicator function denoting whether age group b belongs to the sexually active age groups (15–49 years). The overall contribution was computed by aggregating across both sexes: $\sum_{a \in A} \sum_b m_{t,ab} 1_s(a) \,/\, \sum_{a \in A} \sum_b m_{t,ab}$, with A representing the collection of all groups aged 0–4 or 5–9 years. We also computed the proportion of transmission through sexual contact at time *t* as the expected number of sexually-acquired infections (i.e., infections among high-sexual-activity groups) divided by the total infection counts, in the form of $\sum_a I_{t,a} 1_t(a) / \sum_a I_{t,a}$, where $1_t(\cdot)$ is an indicator function which equals 1 for high-sexual-activity groups and 0 otherwise.

Model fitting

Parameters were estimated using Bayesian inference, where all but the smallpox vaccine effectiveness and the scaling of the NGM were assigned flat prior distributions. We ran five independent chains, each with 2,000 warm-up followed by 5,000 iterations using the No-U-Turn-Sampler algorithm in CmdStan [12]. Convergence was assessed using visual inspection, the Gelman-Rubin convergence diagnostic (with all R-hat values below 1.01), effective sample sizes (ESS, exceeding 400) and the energy Bayesian fraction of missing information (E-BFMI, exceeding 0.3) [13,14]. Posterior draws from all chains were pooled to derive parameter estimates, reported as means and 95% credible intervals (CrIs). All the analyses and visualizations were conducted using R software (version 4.2) [15].

Model fit of the main analysis

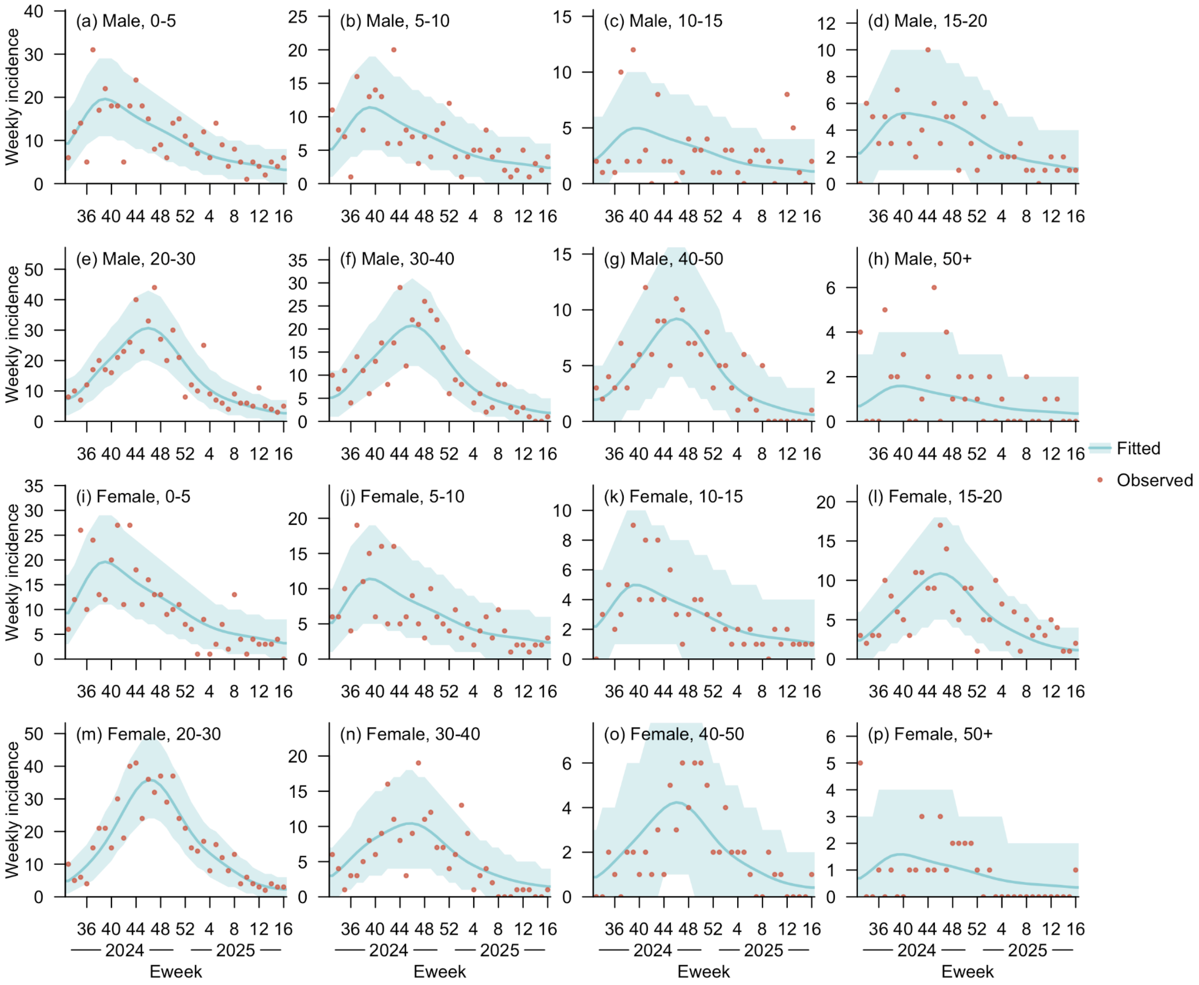

Figure S2. Weekly case counts for each sex and age group in Burundi. Blue lines and shaded areas represent the model's posterior predictive means and the corresponding 95% CrIs, while the red dots represent the reported values from the situation reports. The time range spans from 18 August (Eweek 33) 2024 to 20 April (Eweek 16) 2025. Please note that the y-axis scale differs across the subfigures.

Sensitivity analysis: Impact of spline function degree of freedom on transmission trend estimates

In the main analysis, we used spline functions with a pre-specified six degrees of freedom. Here, we explored models using spline functions with a degree of freedom ranging from four to eight, with the results presented in Figure S3 (where the results for the main analysis are presented in Row 3). Increasing the degree of freedom led to greater variations among the differences between neighbouring values and less consistent trends over time. Nevertheless, the estimated values remained largely similar across the model variants.

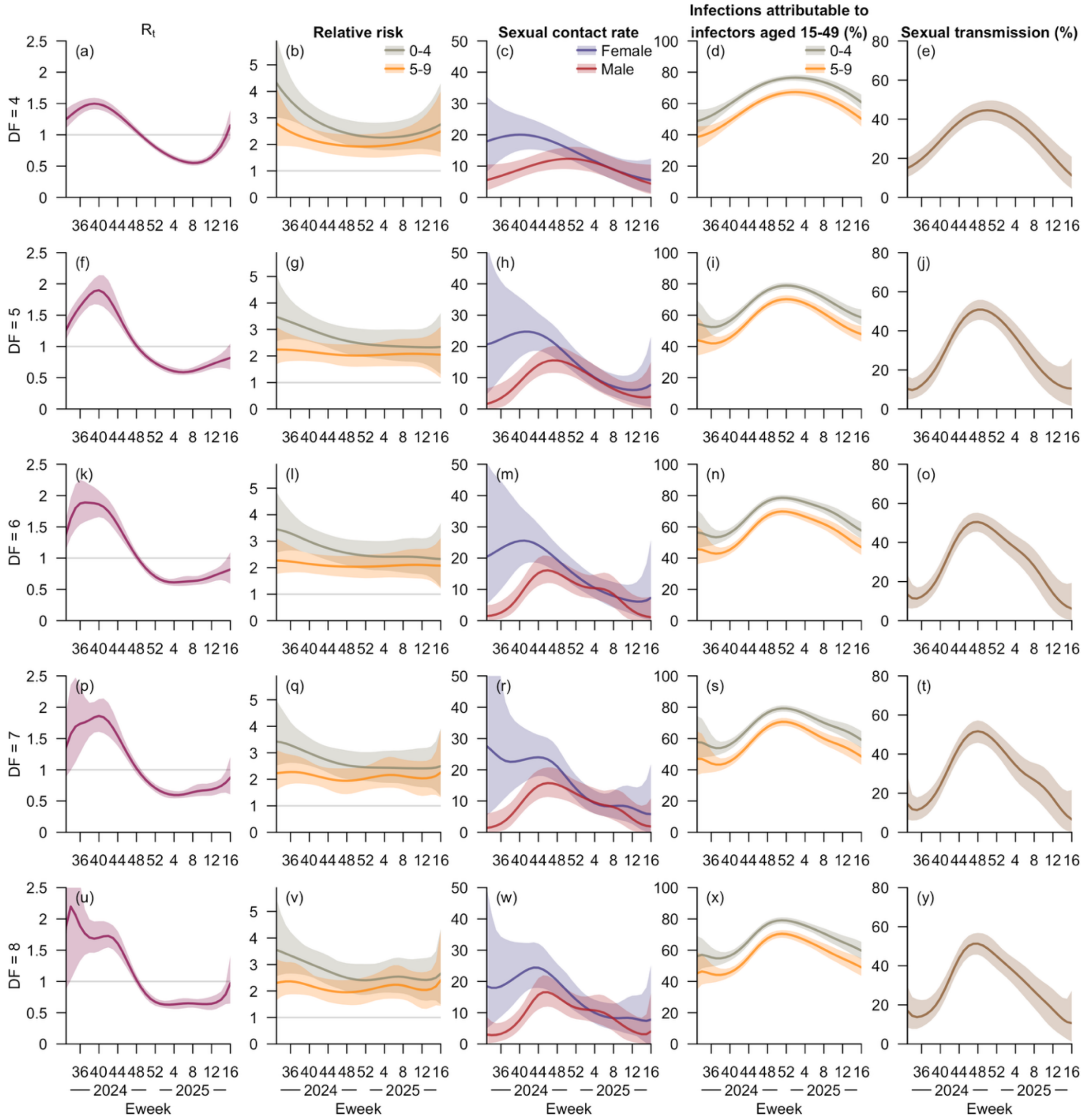

Figure S3. Transmission trend estimates from the model variants using spline functions with varying degrees of freedom (one per row). These include (i) instantaneous effective reproduction number (R_t), (ii) the relative infection risk per infectious contact for children aged 0–4 and 5–9 years compared to the remaining general population, (iii) the sexual

contact rates of the high-sexual-activity groups, (iv) the proportion of infections among children aged 0–4 and 5–9 years attributable to infectors aged 15–49 years, and (v) the proportion of transmission through sexual contact. All the estimates are presented as posterior means (lines) with 95% CrIs (shaded areas). The time frame spans from 18 August (Eweek 33) 2024 to 20 April (Eweek 16) 2025.

Sensitivity analysis: Impact of prior distribution for the scaling of the NGM on transmission trend estimates

We conducted a sensitivity analysis to assess how varying the prior distribution for the scaling of the NGM affected model fits. In the main analysis, we set this prior as a normal distribution truncated from below at zero, $N^{+}(0.15, 0.2^2)$ [10]. For the sensitivity analysis, we explored alternative prior distributions, including a stricter prior $N^{+}(0.15, 0.05^2)$ [16] and a less informative prior $N^{+}(0.15, 0.4^2)$. The model outputs showed that increasing the standard deviation of this prior distribution had a minimal impact on R_t estimates, especially from September 2024 onward (Figure S4, with results for the main analysis in Row 2).

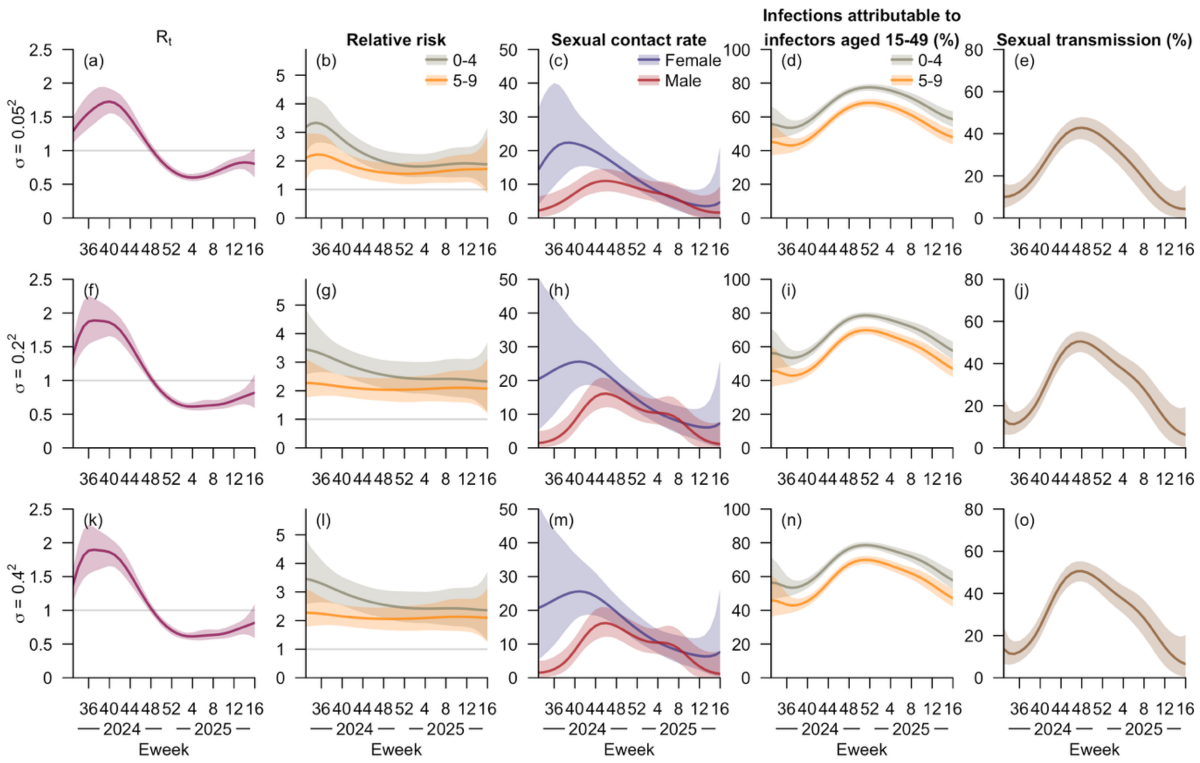

Figure S4. Transmission trends estimates from the model variants which assigned different prior distributions to the scaling of the NGM (one per row, with σ representing the standard deviation parameter in the truncated normal distribution). The estimates include (i) instantaneous effective reproduction number (R_t), (ii) the relative infection risk per infectious contact for children aged 0–4 and 5–9 years compared to the remaining general population, (iii) the sexual contact rates of the high-sexual-activity groups, (iv) the proportion of infections among children aged 0–4 and 5–9 years attributable to infectors aged 15–49 years, and (v) the proportion of transmission through sexual contact. All the estimates are presented as posterior means (lines) with 95% CrIs (shaded areas). The time frame spans from 18 August (Eweek 33) 2024 to 20 April (Eweek 16) 2025.

Sensitivity analysis: Impact of serial interval distribution on transmission trend estimates

In the main analysis, we adopted a Weibull-distributed serial interval with a shape parameter of 1.63, a scale parameter of 18.3, and a right shift of 1 day, leading to a mean of 2.15 weeks and a standard deviation of 0.93 weeks. This distribution was derived from data on two household clade Ia outbreaks in Sudan in 2005 and Central African Republic in 2021–2022. The data reflected transmission of clade Ia outbreaks in household settings, closely resembling the epidemiological context observed in Burundi where household infections were prominent [17]. In this sensitivity analysis, however, we estimated R_t using an alternative distribution with a shape parameter of 2.17, a scale parameter of 6.1, and a right shift of 6 days. This distribution has a mean of 2.15 weeks and a standard deviation of 0.41 weeks, and was based on a hospital-associated clade Ia outbreak in the DRC in 2003, characterised by a smaller sample size and lower variability in serial intervals between symptom onset of infectors and infectees. Both distributions were sourced from a previous study on historical clade Ia outbreaks [18], which, to our knowledge, was the only publication on the serial interval distribution of clade I MPXV available in PubMed as of April 2025. The inferred overall transmission trend remained largely consistent (Figure S5, with results for the main analysis in Row 1).

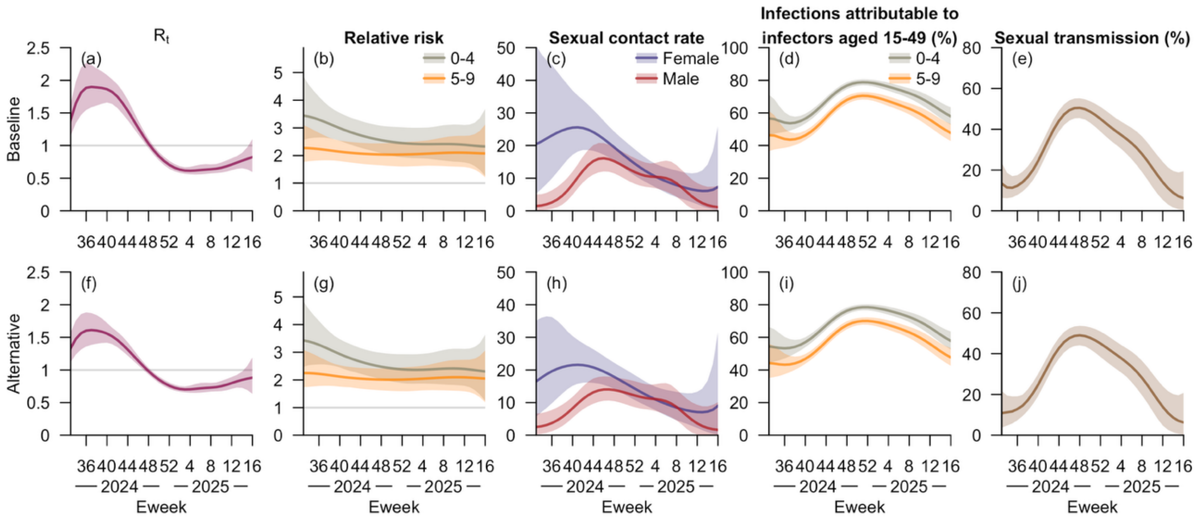

Figure S5. Transmission trend estimates from the model variants adopting different serial interval distributions. The estimates include (i) the relative infection risk per infectious contact for children aged 0–4 and 5–9 years compared to the remaining general population, (ii) the sexual contact rates of the high-sexual-activity groups, (iii) the proportion of infections among children aged 0–4 and 5–9 years attributable to infectors aged 15–49 years, (iv) the proportion of transmission through sexual contact, and (v) instantaneous effective reproduction number (R_t). All the estimates are presented as posterior means (lines) with 95% CrIs (shaded areas). The time frame spans from 18 August (Eweek 33) 2024 to 20 April (Eweek 16) 2025.

Sensitivity analysis: Impact of high-sexual-activity population size on transmission trend estimates

In this sensitivity analysis, we evaluated how varying the size of the high-sexual-activity population would affect the transmission trend estimates. Following the original study [1], we assumed that high-sexual-activity females comprised 2% of individuals aged 15–49 years, based on the estimated sex-worker population size in literature, while in the main analysis the proportion was set at 10%. As presented in Figure S6, the adjustment (Row 2) had a limited impact on the results.

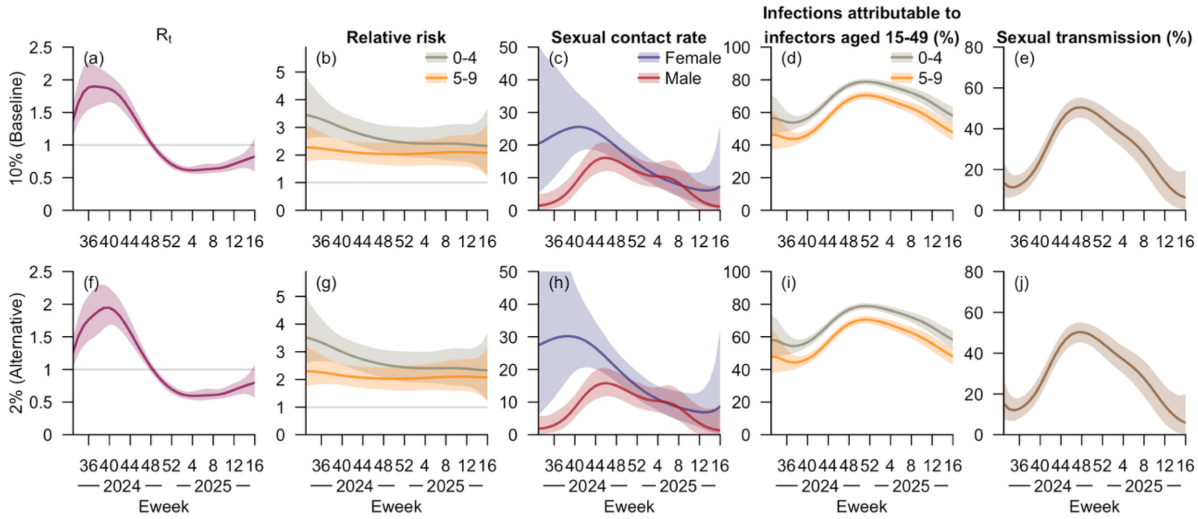

Figure S6. Transmission trend estimates from model variants assuming different proportions of high-sexual-activity individuals among all females aged 15–49 years. The estimates include (i) the relative infection risk per infectious contact for children aged 0–4 and 5–9 years compared to the remaining general population, (ii) the sexual contact rates of the high-sexual-activity groups, (iii) the proportion of infections among children aged 0–4 and 5–9 years attributable to infectors aged 15–49 years, (iv) the proportion of transmission through sexual contact, and (v) instantaneous effective reproduction number (R_t). All the estimates are presented as posterior means (lines) with 95% CrIs (shaded areas). The time frame spans from 18 August (Eweek 33) 2024 to 20 April (Eweek 16) 2025.

Sensitivity analysis: Impact of temporal constraints on transmission trend estimates

In the main analysis, we imposed no constraints on the temporal parameters of the NGM, while in this sensitivity analysis, we evaluated the influence of assuming a non-increasing trend in any of the following three categories of temporal parameters:

(i) the scaling of the temporal trends in transmission of clade Ib MPXV across the entire population,

(ii) the relative infection risk of children aged 0–4 and 5–9 years to the remaining general population, and

(iii) the sexual contact rates for high-sexual-activity males and females.

The results indicated similar R_t estimates across model variants from September 2024 onward and revealed high correlations among the three temporal parameter categories. The inferred decline in R_t between September and December 2024 could be attributed to a reduction in any temporal parameters, while in 2025, imposing a non-increasing constraint on any temporal parameters would result in a non-trivial rise in the estimated values of the remaining unconstrained parameters (Figure S7, with results for the main analysis in Row 1).

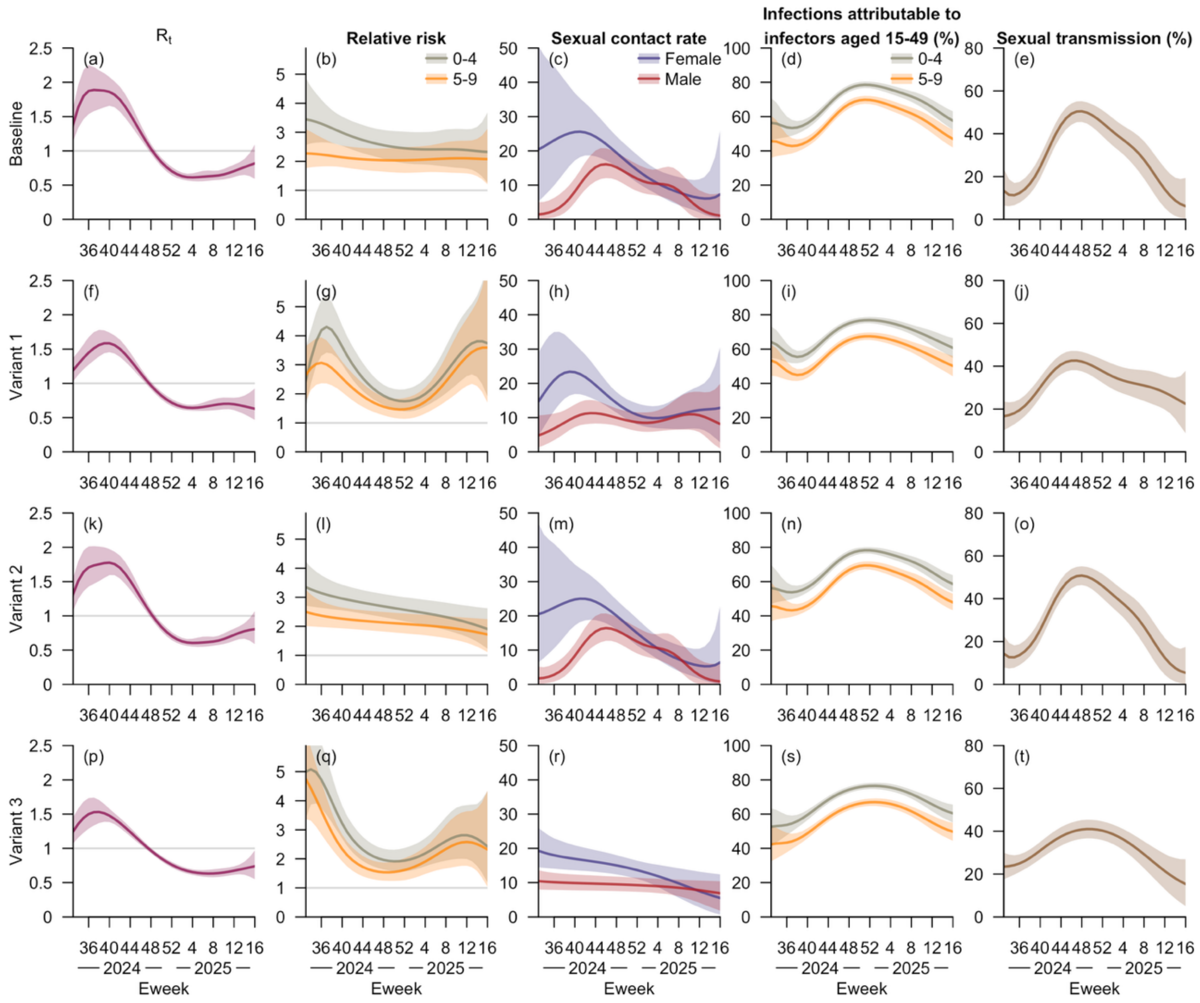

Figure S7. Transmission trends estimates from the model variants assuming a non-increasing trend in various temporal parameters (one per row). The estimates include (i) instantaneous effective reproduction number (R_t), (ii) the relative infection risk per infectious contact for children aged 0–4 and 5–9 years compared to the remaining general population, (iii) the sexual contact rates of the high-sexual-activity groups, (iv) the proportion of infections among children aged 0–4 and 5–9 years attributable to infectors aged 15–49 years, and (v) the proportion of transmission through sexual contact. All the estimates are presented as posterior means (lines) with 95% CrIs (shaded areas). The three model variants each assumed a non-increasing trend in one of the following: (i) the scaling of the temporal trends in transmission of clade Ib MPXV across the entire population, (ii) the relative infection risk of children aged 0–4 and 5–9 years to the remaining general population, and (iii) the sexual contact rates for high-sexual-activity males and females. The time frame spans from 18 August (Eweek 33) 2024 to 20 April (Eweek 16) 2025.

Sensitivity analysis: Impact of sexual mixing pattern on transmission trend estimates

In the main analysis, we assumed sexual contacts of high-sexual-activity individuals were randomly assigned within the subpopulation (i.e., proportionate mixing). However, assortative partner selection may occur, as suggested by Ott et al. in their study of sexual relationships in South Africa [19]. To assess its potential impact on transmission trend estimates, we followed the original study from which our model was adapted [1] and performed this sensitivity analysis to incorporate assortativity by inflating the diagonal elements of the sexual contact matrices $\boldsymbol{S}_{t,X}$ and $\boldsymbol{\Sigma}_{t,X}$:

$$m'_{ij} = (1 - g)m_{ij} + g\delta_{ij} \sum_{k} m_{kj},$$

where $\boldsymbol{M} = (m_{ij})$ denotes the original sexual contact matrix, g is the Gupta's Q quantifying the proportion of partnerships that occur within the same age group, and δ_{ij} is the Kronecker's delta. Following Ott et al., we set g as 0.357 for high-sexual-activity females (contactor) and 0.393 for their male counterparts [19]. As presented in Figure S8, this adjustment (Row 2) only slightly affected estimates of relative risks and sexual contact rates at the beginning of the inference window, where uncertainty was substantial as a result of limited data points to inform parameter values.

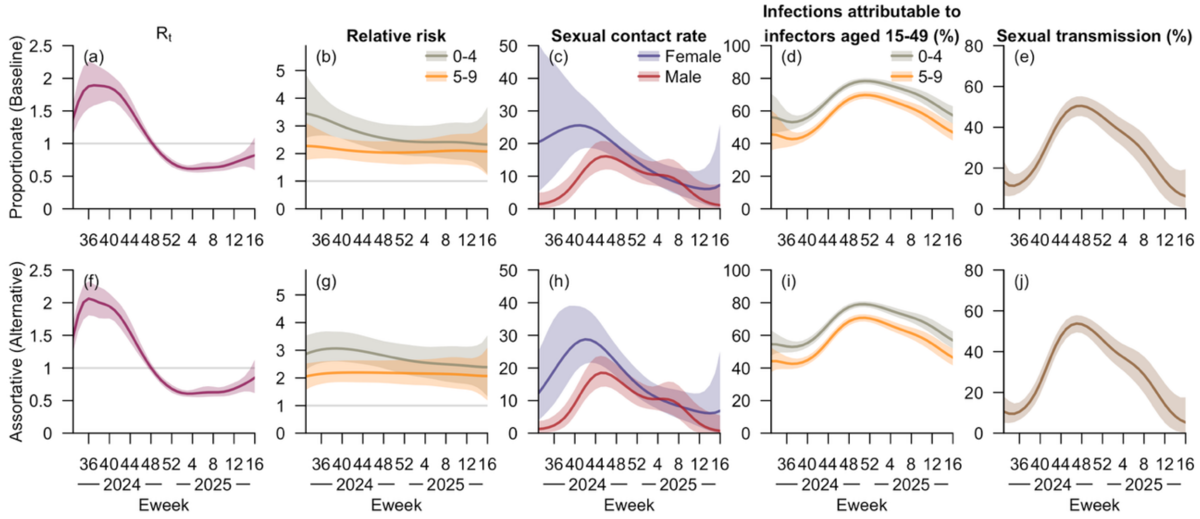

Figure S8. Transmission trend estimates from model variants assuming different sexual contact patterns (with or without assortative mixing) among high-sexual-activity individuals. The estimates include (i) the relative infection risk per infectious contact for children aged 0–4 and 5–9 years compared to the remaining general population, (ii) the sexual contact rates of the high-sexual-activity groups, (iii) the proportion of infections among children aged 0–4 and 5–9 years attributable to infectors aged 15–49 years, (iv) the proportion of transmission through sexual contact, and (v) instantaneous effective reproduction number (R_t). All the estimates are presented as posterior means (lines) with 95% CrIs (shaded areas). The time frame spans from 18 August (Eweek 33) 2024 to 20 April (Eweek 16) 2025.

Sensitivity analysis: Impact of reported case distribution assumption on transmission trend estimates

In the main analysis, we assumed that reported case counts followed a Poisson distribution with mean equal to modelled weekly incidence. To account for potential dispersion, we conducted this sensitivity analysis assuming a Negative Binomial distribution with mean $I_{t,as}$ and variance being $(I_{t,as} + I_{t,as}^2/\phi_{as})$ for age group a and sex s in week t, where ϕ_{as} is the age-sex-specific dispersion parameter. The resulting posterior predictive distributions of the weekly case counts were similar to those generated by the model using a Poisson distribution in the main analysis (Figure S9). Likewise, the estimated relative risks and sexual contact rates were generally consistent across the two models (Figure S10).

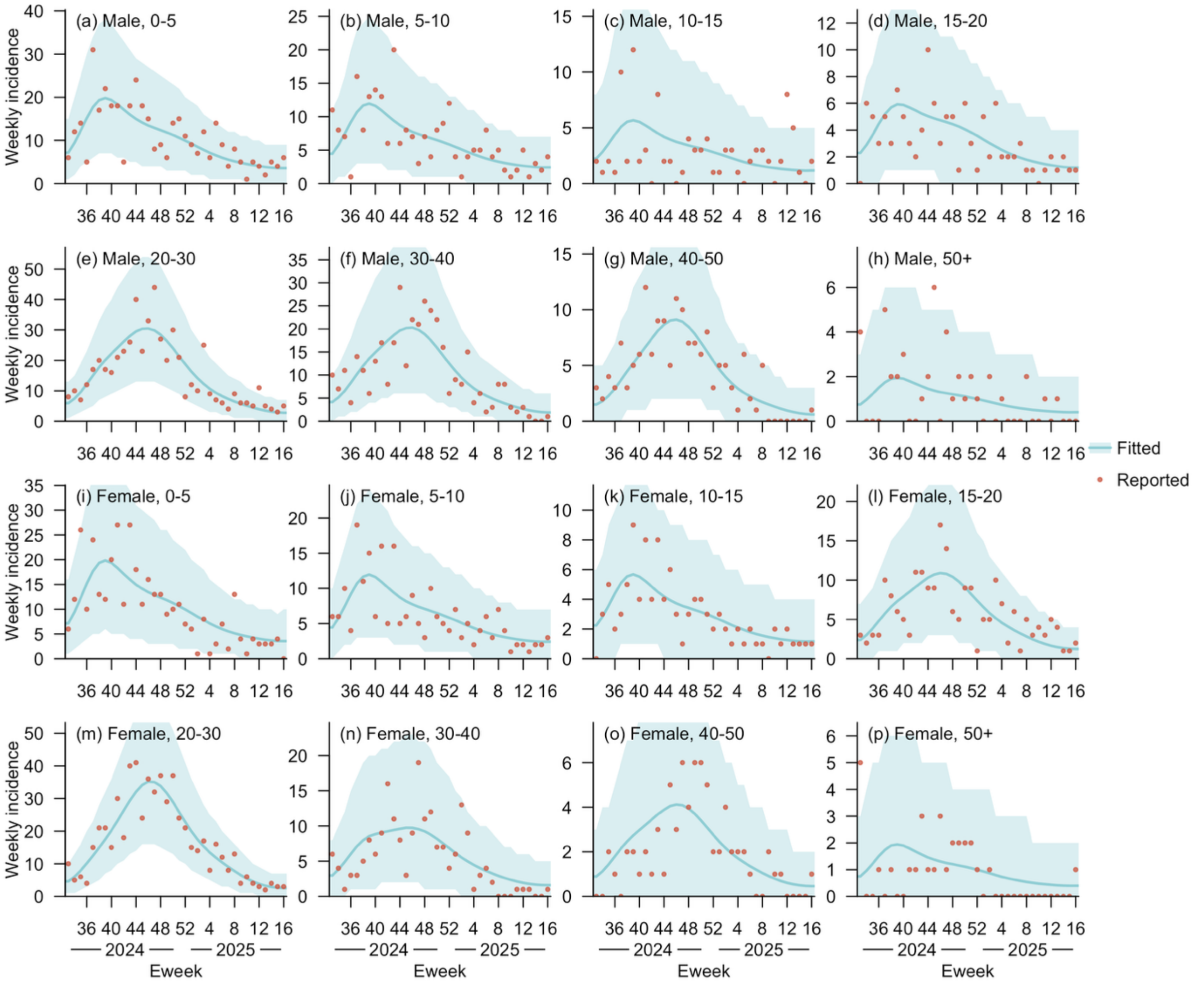

Figure S9. Weekly case counts for each sex and age group in Burundi, obtained from the model assuming a Negative Binomial distribution for weekly case counts. Blue lines and shaded areas represent the model's posterior predictive means and the corresponding 95% CrIs, while the red dots represent the reported values from the situation reports. The time range spans from 18 August (Eweek 33) 2024 to 20 April (Eweek 16) 2025. Please note that

the y-axis scale differs across the subfigures.

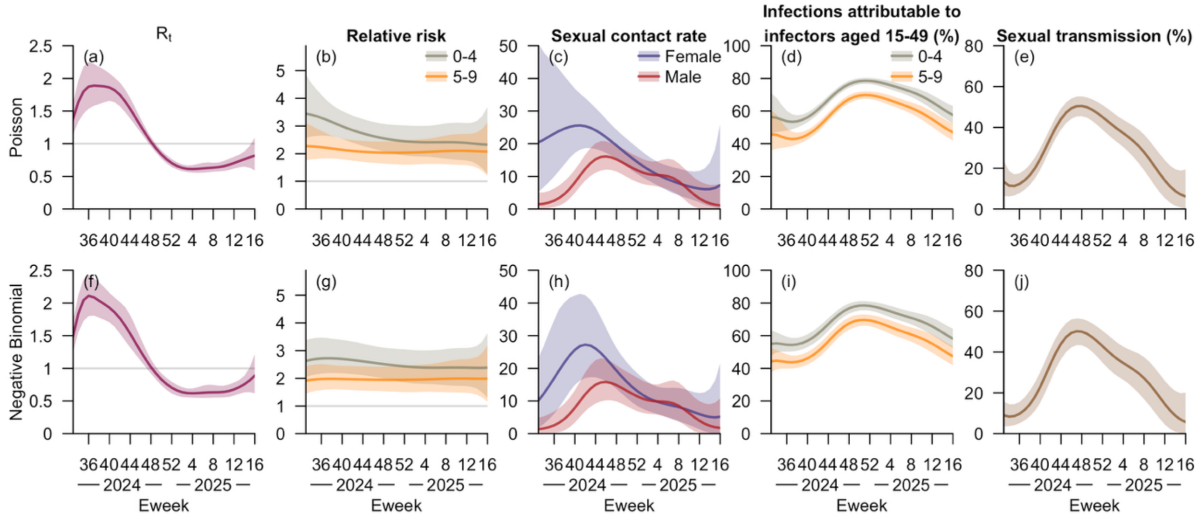

Figure S10. Transmission trend estimates from model variants using a Poisson (Row 1, main analysis) distribution or a Negative Binomial (Row 2, sensitivity analysis) distribution for reported case counts. The estimates include (i) the relative infection risk per infectious contact for children aged 0–4 and 5–9 years compared to the remaining general population, (ii) the sexual contact rates of the high-sexual-activity groups, (iii) the proportion of infections among children aged 0–4 and 5–9 years attributable to infectors aged 15–49 years, (iv) the proportion of transmission through sexual contact, and (v) instantaneous effective reproduction number (R_t). All the estimates are presented as posterior means (lines) with 95% CrIs (shaded areas). The time frame spans from 18 August (Eweek 33) 2024 to 20 April (Eweek 16) 2025.

References

1. Murayama H, Asakura TR, Dickens BL, Foo JH, Jin S, Mukadi PK, et al. Roles of community and sexual contacts as drivers of clade I mpox outbreaks [Internet]. medRxiv; 2024 [cited 2024 Dec 19]. p. 2024.10.15.24315554. Available from: https://www.medrxiv.org/content/10.1101/2024.10.15.24315554v1

2. Taube JC, Rest EC, Lloyd-Smith JO, Bansal S. The global landscape of smallpox vaccination history and implications for current and future orthopoxvirus susceptibility: a modelling study. Lancet Infect Dis. 2023 Apr 1;23(4):454–62.

3. Fenner F, Henderson DA, Arita I, Jezek Z, Ladnyi ID, Organization WH. Smallpox and its eradication [Internet]. World Health Organization; 1988 [cited 2025 Aug 8]. Available from: https://iris.who.int/handle/10665/39485

4. WHO. Multi-country outbreak of mpox, External situation report #51 [Internet]. 2025 [cited 2025 May 28]. Available from: https://www.who.int/publications/m/item/multi-country-outbreak-of-mpox--external-situation-report--51---29-april-2025

5. Melegaro A, Fava ED, Poletti P, Merler S, Nyamukapa C, Williams J, et al. Social Contact Structures and Time Use Patterns in the Manicaland Province of Zimbabwe. PLOS ONE. 2017 Jan 18;12(1):e0170459.

6. Inungu JN, Kandala BFN, Atungale AM. Prevalence of HIV and Syphilis and Related Risk Behaviors Among Female Sex Workers in the Democratic Republic of the Congo. Eur J Environ Public Health. 2022 June 25;6(2):em0114.

7. Pareek A, Singhal R, Pareek A, Chuturgoon A, Apostolopoulos V, Chattu VK. Global spread of clade Ib mpox: a growing concern. Lancet Microbe. 2025 Apr 8;101132.

8. Kibungu EM, Vakaniaki EH, Kinganda-Lusamaki E, Kalonji-Mukendi T, Pukuta E, Hoff NA, et al. Clade I–Associated Mpox Cases Associated with Sexual Contact, the Democratic Republic of the Congo. Emerg Infect Dis. 2024 Jan;30(1):172–6.

9. Burundi Public Health Emergency Operation Center. COUSP [Internet]. 2025 [cited 2025 June 9]. Available from: https://www.cousp-minisante.gov.bi/

10. Beer EM, Rao VB. A systematic review of the epidemiology of human monkeypox outbreaks and implications for outbreak strategy. PLoS Negl Trop Dis. 2019 Oct 16;13(10):e0007791.

11. Akter F, Hasan TB, Alam F, Das A, Afrin S, Maisha S, et al. Effect of prior immunisation with smallpox vaccine for protection against human Mpox: A systematic review. Rev Med Virol. 2023;33(4):e2444.

12. Jonah Gabry, Rok Češnovar, Andrew Johnson, Steve Bronder. cmdstanr: R Interface to "CmdStan" [Internet]. 2025 [cited 2025 June 9]. Available from: https://mc-stan.org/cmdstanr/

13. Vehtari A, Gelman A, Simpson D, Carpenter B, Bürkner PC. Rank-Normalization, Folding, and Localization: An Improved $\hat{R}$ for Assessing Convergence of MCMC (with Discussion). Bayesian Anal. 2021 June;16(2):667–718.

14. Stan Development Team. How to Diagnose and Resolve Convergence Problems [Internet]. Stan: Software for Bayesian Data Analysis. 2025 [cited 2025 Aug 8]. Available from: https://mc-stan.org/learn-stan/diagnostics-warnings.html

15. R Core Team. R: A Language and Environment for Statistical Computing [Internet]. Vienna, Austria: R Foundation for Statistical Computing; 2025. Available from: https://www.R-project.org/

16. Otshudiema JO, Nkengurutse L, Kamwenubusa G, Diallo I, Sibomana A, Nsavyimana O, et al. Epidemiological Characteristics and Transmission Dynamics of Mpox in Bujumbura, Burundi: A Prospective Cohort Study [Internet]. Rochester, NY: Social Science Research Network; 2025 [cited 2025 May 22]. Available from: https://papers.ssrn.com/abstract=5144118

17. Nkengurutse L, Otshudiema JO, Kamwenubusa G, Diallo I, Nsavyimana O, Mbonicura JC, et al. Clinical Predictors and Determinants of Mpox Complications in Hospitalized Patients: A Prospective Cohort Study from Burundi. Viruses. 2025 Apr;17(4):480.

18. Marziano V, Guzzetta G, Longini I, Merler S. Epidemiologic Quantities for Monkeypox Virus Clade I from Historical Data with Implications for Current Outbreaks, Democratic Republic of the Congo. Emerg Infect Dis. 2024 Oct;30(10):2042–6.

19. Ott MQ, Bärnighausen T, Tanser F, Lurie MN, Newell ML. Age-gaps in sexual partnerships: seeing beyond 'sugar daddies.' AIDS Lond Engl. 2011 Mar 27;25(6):861–3.
